# Spatiotemporal Analysis of Invasive Meningococcal Disease, Germany

**DOI:** 10.3201/eid1211.060682

**Published:** 2006-11

**Authors:** Johannes Elias, Dag Harmsen, Heike Claus, Wiebke Hellenbrand, Matthias Frosch, Ulrich Vogel

**Affiliations:** * University of Würzburg, Würzburg, Germany;; † University Hospital, Muenster, Germany;; ‡ Robert Koch-Institute, Berlin, Germany

**Keywords:** Neisseria meningitidis, meningococcal infections, cluster analysis, bacterial typing techniques, research

## Abstract

Meningococci can cause clusters of disease. Specimens from 1,616 patients in Germany obtained over 42 months were typed by serogrouping and sequence typing of PorA and FetA and yielded a highly diverse dataset (Simpson's index 0.963). A retrospective spatiotemporal scan statistic (SaTScan) was applied in an automated fashion to identify clusters for each finetype defined by serogroup variable region (VR) VR1 and VR2 of the PorA and VR of the FetA. A total of 26 significant clusters (p<0.05) were detected. On average, a cluster consisted of 2.6 patients. The median population in the geographic area of a cluster was 475,011, the median cluster duration was 4.0 days, and the proportion of cases in spatiotemporal clusters was 4.2%. The study exemplifies how the combination of molecular finetyping and spatiotemporal analysis can be used to assess an infectious disease in a large European country.

## Abstract 2

Meningococcal disease clustering was found by DNA sequence–based finetyping and cluster detection software.

Infection with meningococci in a susceptible human host can involve septicemia and meningitis, which are referred to as invasive meningococcal disease (IMD). IMD generates public concern and panic because of its often lethal outcome, its propensity to affect the young, and its occasional appearance in clusters. Meningococci are highly variable bacterial pathogens, as shown by a multitude of different sequence types identified by multilocus sequence typing (MLST) (*1*) and by antigen sequence typing of the outer membrane proteins such as PorA (*2*) and FetA (*3*).

Use of DNA sequence-based typing has several advantages over serotyping: information is reproducible and portable, most isolates are typeable, and culture-independent typing is possible. The consistent use of DNA sequence typing at the German National Reference Center for Meningococci (NRZM) since December 2001 has resulted in an extensive database containing a large number of unambiguously typed isolates. We define the term finetype as the antigenic profile of a meningococcal strain consisting of the serogroup, the sequence types of the variable regions (VRs) VR1 and VR2 of the PorA, and the sequence type of the immunodominant VR of FetA. The European Monitoring Group on Meningococci recommended in 2005 that PorA sequence typing be implemented as a standard typing method in all participating countries by 2007.

A meningococcal disease cluster is regarded as an aggregation of cases caused by the same bacterial strain closely grouped in space and time. While most cases of IMD appear in a sporadic fashion in industrialized countries, coprimary (i.e., occurring within 24 hours) and secondary cases occur regularly (*4*), as shown in institutional and household surveys (*5*). A community outbreak (*6*) represents an excess of incidence in a defined geographic area or population, in which direct links between cases are not always readily apparent. In most instances, detection of increases in case counts within defined spatial and temporal boundaries, for lack of more objective methods, must rely on the attentiveness of public health officials (*7*). Computer-assisted spatiotemporal cluster analyses might help identify and statistically evaluate increased instances of meningococcal disease, thus providing valuable information for further public health investigation and intervention.

Many methods have been developed for cluster analysis (*8*). A stochastic model has been applied to predict outbreaks of meningococcal disease in closed communities such as military cohorts (*9*). Hoebe et al. used space-time nearest neighbor analysis to statistically evaluate clusters of IMD in the Netherlands (*10*). One of the most widely used software packages for cluster analysis is SaTScan, which was developed by Martin Kulldorff (National Cancer Institute, Bethesda, MD, USA) and Farzad Mostashari (New York Department of Health and Mental Hygiene, New York, NY, USA) (*11*). In infectious disease epidemiology, SaTScan has been used to study listeriosis (*12*), methicillin-resistant Staphylococcus aureus infection (*13*), gonorrhea (*14*), West Nile fever (*15*), Creutzfeldt-Jakob disease (*16*), bovine respiratory syncytial virus (*17*), and pediatric pneumonia (*18*). Furthermore, national bioterrorism syndromic surveillance (*19*) and public health systems (*20**,**21*) rely on the use of this program. We applied SaTScan to a rigorously typed strain collection to identify and quantify finetype-specific clusters of cases of IMD in a large central European country with endemic meningococcal disease.

## Materials and Methods

### Data Collection

Meningococcal strains and culture-negative specimens obtained from patients with IMD are referred to NRZM by regional laboratories and hospitals for finetyping and, where applicable, antimicrobial drug resistance testing. Data are managed at NRZM by using a Microsoft Access database (Microsoft Corp., Redmond, WA, USA) that provides a user-friendly entry and retrieval surface. From December 1, 2001, to June 1, 2005, meningococci were detected from a normally sterile site in 1,828 patients; 1,616 patients with complete typing data and available residential postcode were included in the analysis. In 46 (2.8%) of these 1,616 cases, only clinical material (i.e., cerebrospinal fluid or serum) was analyzed. Of the remaining patients, no postcodes were available, no meaningful geographic information could be extracted from the postcode provided by the submitting laboratory, or finetyping could not be performed. Although the first 2 causes were outside the realm of NRZM, the last cause was mainly due to limitations of DNA sequence typing of meningococcal DNA from native samples such as cerebrospinal fluid or blood. A recent capture-recapture analysis for 2003 showed that NRZM processes samples from »65% of all cases estimated to occur in Germany (*22*). Underreporting to the NRZM occurs because submission of data by regional laboratories is voluntary, whereas reporting to the Robert Koch-Institute is statutory. For assessment of the systematic bias introduced by reporting behavior, see Adjustment for Potential Confounders in the Results section.

During the time of the study, no general recommendation existed in Germany for serogroup C conjugate vaccination. Two limited vaccination campaigns were initiated after observation of clusters 11 and 19 reported herein (Table).

**Table:** Clusters of invasive meningococcal disease detected by SaTScan analysis, Germany, December 2001–June 2005

Cluster	Finetype	Cases	States (counties)*	Population	Year	Duration (d)	p value†	p_age_ value‡
1	Y:P1.5–2,10–28:F4–1	2	BY (1)	213,603	2002	21	0.003	0.003
2	B:P1.7–2,4:F3–3	2	NI (2)	2,286,265	2002	4	0.002	0.003
3	B:P1.18–1,30:F3–3	2	HH (1), NI (1)	3,096,084	2002	23	0.023	0.028
4	B:P1.5–1,2–2:F1–5	2	NI (1)	206,304	2002	18	0.006	0.004
5	B:P1:18,25–1:F5–1	2	TH (1)	142,595	2003	16	0.011	0.01
6	B:P1.5–2,10:F5–1	3	HE (1), RP (1)	2,394,079	2003	17	0.026	0.023
7	C:P1.5,2:F1–7	2	SL (1)	349,102	2003	3	0.035	0.033
8	B:P1.7,16:F5-X§	2	BY (2)	913,368	2003	10	0.028	0.025
9	C:P1.22,9:F3–3	3	NW (2), RP (1)	5,441,714	2003	2	0.002	0.002
10	C:P1.5,2:F3–3	4	BB (1), SN (1)	339,185	2003	18	0.004	¶
11	C:P1.5–1,10–8:F3–6	2	NW (1)	429,832	2003	4	0.008	0.011
12	C:P1.5–1,10–8:F4–1	2	BW (1)	134,407	2003	13	0.028	0.043
13	C:P1.5,2:F1–1	2	NW (2)	860,407	2003	1	0.037	0.032
14	B:P1.5–1,2–2:F1–14	2	MV (1)	120,959	2003	<1	0.001	0.001
15	C:P1.5,2:F5–8	3	NW (1), RP (2)	2,768,981	2003	1	0.001	0.005
16	W135:P1.5,2:F1–1	2	BW (2)	2,761,536	2003	2	0.044	*0.056*
17	B:P1.7,16:F3–3	2	MV (1)	52,994	2004	4	0.041	0.037
18	C:P1.5,2:F5–8	2	BY (1)	42,665	2004	4	0.001	0.006
19	C:P1.5,2:F3–6	2	BY (1)	148,953	2004	4	0.007	0.01
20	B:P1.5–1,2–2:F5–8	2	HE (1), NI (1)	3,076,129	2004	<1	0.002	0.002
21	B:P1.7–2,4:F5–1	2	BY (1)	243,545	2004	<1	0.003	0.003
22	B:P1.7–2,13–9:F5–5	2	NW (1)	239,183	2005	4	0.001	0.001
23	B:P1.7–2,16:F3–3	5	TH (3)	2,399,167	2005	24	0.001	0.001
24	B:P1.7–2,4:F1–5	10	NW (3), RP (1), SL (1)	1,524,166	2005	22	0.001	0.001
25	C:P1.22,14:F3–3	2	BB (1), SN (1)	1,512,043	2005	5	0.012	0.01
26	C:P1.5,2:F3–6	2	BY (2)	520,190	2005	7	0.018	0.02

*BY, Bavaria; NI, Lower Saxony; HH, Hamburg; TH, Thuringia; HE, Hesse; RP, Rhineland-Palatinate; SL, Saarland; NW, NorthRhine-Westphalia; BB, Brandenburg; SN, Saxony; BW, Baden-Wuerttemberg; MV, Mecklenburg-West-Pomerania. †p values from the unadjusted 42-mo scan. ‡p values from the age-adjusted scan (p value >0.05 is shown in *italics*). §FetA type 5-X has not yet been assigned. ¶Not detected because of missing date of birth in 1 case (see text).

### Meningococcal Typing

Serogrouping of meningococcal isolates was accomplished by slide agglutination with monoclonal antibodies NmA 932, NmB 735, NmW135 1509, and NmY 1938 (Chiron-Behring, Marburg, Germany) and Neisseria meningitidis group C agglutinating sera (Remel, Lenexa, KS, USA). Culture-independent genotyping of meningococci was performed by amplification of polysialyltransferase genes specific to the serogroups B, C, W-135, and Y (*23*). Finetyping was accomplished by amplification and DNA sequencing of VR1 and VR2 of the porA gene encoding PorA and the VR of the fetA gene encoding the FetA protein (*2**,**3*). A finetype is expressed by the antigenic profile serogroup: P1.VR1,VR2:FVR1, where P1 is PorA and F is FetA. Deduced amino acid sequences were compared with entries in databases accessible at www.neisseria.org, which is curated by Keith Jolley (Oxford, UK) and Ian Feavers (Potters Bar, UK). Sequence data were analyzed with LASERGENE sequence-analysis software (DNASTAR, Madison, WI, USA) and TraceEdit Pro (Ridom, Würzburg, Germany).

DNA from culture-negative cerebrospinal fluid, blood, or serum was extracted by using the QIAamp DNA blood mini kit (Qiagen, Hilden, Germany). Sensitive PCR protocols have been developed and validated to amplify serogroup-specific polysialyltransferase genes, and the variable regions of porA and fetA from culture negative specimen (data not shown). The discriminatory power of the typing methods was assessed by using the numeric index of the discriminatory ability derived from the Simpson index of diversity (*24*). The 95% confidence intervals (CIs) for the numeric indices were calculated as described by Grundmann et al. (*25*).

### SaTScan Spatiotemporal Analysis

Information about SaTScan version 5.1.1 software is available at http://www.satscan.org. The program applies a likelihood function to circular windows originating at defined locations of increasing size and compares observed and expected case numbers inside and outside the scan window to detect clusters that are least likely to have occurred by chance. The statistical significance for each cluster is obtained through Monte Carlo hypothesis testing, i.e., results of the likelihood function are compared for a large number of random replications of the dataset generated under the null hypothesis. In this study, cases were assumed to be Poisson distributed in each location and the program's space-time scan statistic was applied. A user-friendly interface was programmed in Visual Basic for Applications, operating from within the database of NRZM: it handled the automatic data transfer to SaTScan and the creation of concise reports after completion of the analysis. Duplicate samples were identified and excluded automatically before the scan. The date of specimen sampling was defined as time of illness and the county of residence, derived from the postcode, was used as place. The date of submission to NRZM was used if the date of sampling could not be determined (in 2.5% of all cases). Spatiotemporal scanning was initiated at the centroids of the 439 German counties. These represent intermediate administrative units between the German states and the local levels (Gemeinden) and vary in size and population (there are 35,846–3,392,425 inhabitants/county). A county, which can also be a larger town, is the smallest public health unit. The maximum spatial cluster size was chosen to correspond to 7% of the German population (5,777,219). The maximal temporal cluster size was set to 30 days. Age-adjusted scans were performed with age groups >18 years of age and <18 years of age as a covariate. Adjustments for missing data were made according to the program's user manual to account for counties that did not refer samples to NRZM yet reported cases to the Robert Koch-Institute (the central federal German institution responsible for disease control and prevention). Clusters were considered significant for p values £0.05. Each finetype was analyzed separately.

### Geographic Maps

Latitude and longitude coordinates (map date WSG 84) of the centroids of each German county and age-stratified census data of the year 2003 were obtained from GfK Macon (Waghäusel, Germany). Maps were generated and edited with the programs Regiograph 8 (GfK Macon) and Fireworks MX Macromedia (Adobe Systems Inc., San Jose, CA, USA).

## Results

### Finetyping of Meningococci

**Figure 1:**
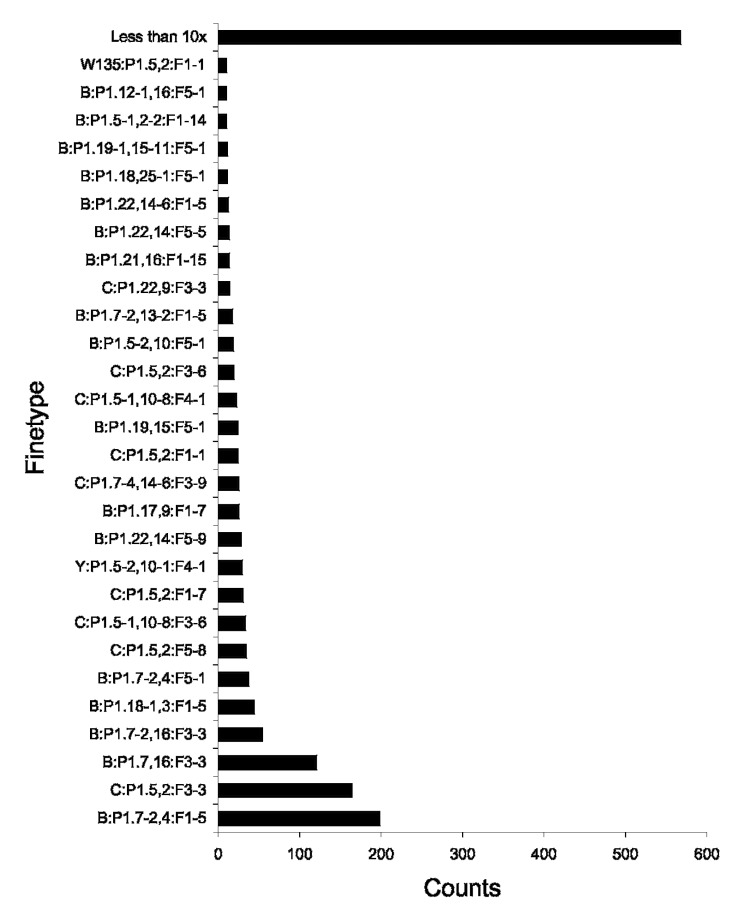
Distribution of 383 finetypes included in the present study (1,616 patients). The most common finetype (B:P1.7–2,4:F.1–5) accounted for 12.3% of the cases.

We analyzed data from 1,616 patients who contracted IMD in Germany from December 2001 through June 2005. Geographic data were inferred successfully from the 5-digit postcode for all patients. Complete finetyping results (serogroup: P1.VR1,VR2:FVR1) were available for all patients. A rank-abundance plot of all finetypes found during the study period indicated the diversity of pathogenic meningococci (Figure 1). The proportion of persons <18 years of age was 74.1%. The serogroup distribution was 65.7%, 28.8%, 3.1%, 1.9%, and 0.3% for serogroups B, C, Y, W135, and 29E, respectively. One case each was caused by serogroups A and Z and a capsule null locus isolate (*26*). A total of 33, 69, and 66 variants of PorA VR1, PorA VR2, and FetA, respectively, were identified. The number of unique combinations of serogroups PorA VR1, PorA VR2, and FetA VR was 383. FetA typing increased the number of finetypes 2.3-fold compared with the number of serogroup PorA VR1 and PorA VR2 combinations alone (167 distinct types). After removing all but 1 strain per cluster from the complete set of data, we determined the numeric index of the discriminatory ability of our typing procedure. Its value for serogroup:PorA typing was 0.930 (95% CI 0.923–0.937) compared with 0.963 (95% CI 0.959–0.968) for serogroup:PorA:FetA typing. The addition of FetA typing increased the discriminatory power of our typing procedure.

### Cluster Analysis

**Figure 2:**
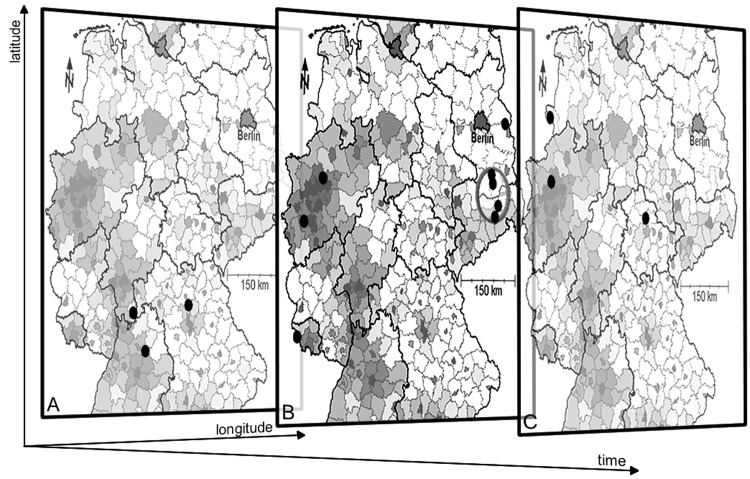
Retrospective identification of a cluster (cluster 10) of the finetype C:P1.5,2:F3–3 in 3 temporal planes using SaTScan (*11*). Planes A, B, and C represent consecutive temporal windows of 30 days in 2003. Cases with the finetype in question are shown by dark ovals defined by the dimensions longitude, latitude, and time. Although planes A and C do not show spatial clustering, plane B shows an accumulation of 4 cases in 2 counties within a circle encompassing a population of 339,185 (radius 28.78 km, p = 0.004; marked by a gray oval). Counties of Germany are shaded according to their population density (darker indicates a higher population density).

SaTScan analysis was applied separately to each finetype present more than once in the historic dataset (134 finetypes) to identify clustering of meningococcal disease in space and time to a degree beyond that expected by chance alone (Table). Analysis identified 26 clusters that included 68 cases (4.2% of all cases). The maximum number of patients per cluster was 10. The median duration of the clusters was 4.0 days (range <1–24 days) and the median interval between the first and the second case was also 4.0 days (range <1–23 days). The median population within the scan windows imposed by SaTScan was 475,011 (range 42,665–5,441,714). In 76.9% of the clusters, only 2 patients were assigned to a cluster. Figure 2 shows the retrospective identification of a cluster of the finetype C:P1.5,2:F3–3 (cluster 10, Table).

### Adjustment for Potential Confounders

The following potentially confounding variables were evaluated. Scans were adjusted for age because most cases occurred in persons <18 years of age (74.1%), the proportion of which was nonhomogeneously distributed per county (range 12%–26%, data not shown). The date of birth was missing for 8 patients (0.4%), who were therefore excluded from the adjusted scan. Only 1 cluster identified in the nonadjusted scan was assigned a p value >0.05 after age-adjustment (cluster 16, Table). Age did not substantially confound the results of the scan.

Underreporting to NRZM by some counties was addressed. For cases submitted in 2003, we performed SaTScan analysis excluding 66 counties identified as having cases of IMD reported to the Robert Koch-Institute but not to NRZM. The adjusted scan resulted in slight changes in the p values of detected clusters compared with the unadjusted scan but detected the same clusters (data not shown).

## Discussion

Our study quantified the proportion of IMD cases that occurred in clusters in a large central European country in a period of 42 months. The following technical prerequisites permitted this large-scale investigation: free availability of the cluster detection software SaTScan, implementation of an automatic data transfer between our database and SaTScan, availability of data regarding time and place of occurrence of IMD, and state-of-the-art highly discriminatory finetyping techniques for the infectious agent in question.

The proportions of different antigenic profiles of meningococci represented by finetypes are subject to temporal and spatial changes because of constant interaction with host immunity (*27*). Compared with sequence types obtained by MLST, PorA and FetA finetypes are expected to fluctuate to a greater extent over time. The application of PorA and FetA finetyping for cluster analysis is warranted because of its consistency within clusters appearing for days or weeks. Exceptions exist, e.g., an outbreak of meningococci differing in its ability to express porA has been reported (*28*). However, this phenomenon would not affect cluster detection by our approach because we used sequence-based typing. We detected 1 epidemiologically related cluster in which strains did not uniformly contain the fetA gene (cluster 11, Table). In general, we believe that these examples represent exceptions. MLST is probably not suitable for a timely national laboratory surveillance of clusters of meningococcal disease because of its considerable requirement for resources. The dataset reported here is the first comprehensive application of FetA typing, which was introduced as an alternative marker for meningococci in 2003 (*3*). FetA typing has proven to be reliable and easy to use. Moreover, it increased the discriminatory power of our typing procedure. The results of this study thus support the extended use of FetA sequence typing.

SaTScan was chosen because it is the most thoroughly evaluated software for detecting spatiotemporal clusters of infectious diseases. Application of a Poisson distribution to the epidemiology of a rare disease such as IMD is appropriate, although in practice the null hypothesis (i.e., complete spatial randomness) cannot be expected to be true even if no clusters of disease exist for a given spatiotemporal expanse. SaTScan serves as a tool that directs the attention of its user to anomalous case distributions. The p values are automatically adjusted for the multiple testing stemming from repeated evaluations of different potential clusters during hypothesis testing.

Hoebe et al. applied a global clustering test (space-time nearest neighbor analysis) to different serosubtypes of meningococci and found statistical evidence for clustering in 6 of 25 clusters reported by the Dutch Inspectorate of Health Care (*10*). However, connections of >2 cases could not be demonstrated beyond chance. Since only phenotypic typing was performed, the analysis was restrained by a considerable proportion of nontypeable isolates. In contrast to the Dutch study, we included both viable strains and sterile specimens in our investigation. Only fully-typed strains and DNA were evaluated for the existence of spatiotemporal clusters. Since we used a cluster detection test rather than a global clustering test, we were able to pinpoint clusters of meningococcal disease even for rare finetypes in space and time. The detection of the presented clusters as such did not depend on the attentiveness of public health workers. Spatiotemporal proximity could be shown for up to 10 patients (cluster 24, Table). Similar to findings of other studies (*29*), most clusters had only 2 patients.

The temporal settings of our scans were defined according to results from earlier retrospective cluster studies. An American analysis (*30*) found that 73% of secondary cases appeared <14 days after the index case. In France, 72% of secondary cases occurred in the first week after the first case (*5*). A British survey determined the median intervals between the index case and the second case to be 1.5, 5, or 23 days, depending on the setting of the cluster (household, school, or university) (*29*). Thus, a maximal temporal window of 30 days should detect most of the existing clusters, although the time between the first and the second case may rarely exceed this temporal limit.

Two spatially confined immunization campaigns were conducted after outbreaks of IMD caused by ET-15 meningococci. Our analysis detected 2 clusters representing each of them (clusters 11 and 19). The first campaign targeted a single county in North-Rhine-Westphalia; the second one comprised only a few boroughs within a county in Bavaria (*31*). Theoretically, spatially uneven vaccine coverage could introduce a regional bias into our analysis, e.g., by creating areas with low carriage rates (herd protection). Because of the low number and confined nature of the campaigns, a possible bias was not assessed but is likely negligible. A general recommendation for vaccination against serogroup C disease in Germany was made in 2006, i.e., after this study. Previously, only the State of Saxony had a general recommendation. However, reimbursement of costs was not guaranteed there, and precise numbers of vaccinees are not available. One also has to consider that most cases of meningococcal disease in Germany are not preventable by vaccination.

Most clusters of meningococcal disease occur in households or social units that provide educational services in workplaces, and through other forms of social interaction. To curtail computing time while providing an acceptable geographic resolution, counties represented the smallest geographic units in our analysis. The variable size of the counties leads to fewer possible cluster locations evaluated in the area of large counties. The increased geographic aggregation in larger counties may also reduce the power to detect small clusters. However, since maximal spatial cluster size was chosen to correspond to 7% of the German population, detection of clusters spanning neighboring counties was warranted in all positions of our grid (e.g., Berlin's population plus that of the counties encircling it comprise <7% of the population of Germany). Performing cluster analyses on the basis of the patient's residence may not always reflect the area of the social network where acquisition of IMD occurred. Infection might be contracted at locations other than the one suggested by the postcode, e.g., at gatherings outside the county of residence. Thus, a few supraregional clusters might have been missed by our approach.

The proportion of patients involved in clusters in Germany was 4.2% (95% CI 3%–5.3%). Interpretation of this figure must consider that not all cases of IMD are assessed at NRZM. Conversely, all clusters reported herein were verified by finetyping. In 42 months, 26 clusters were detected. In France, 28 clusters of meningococcal disease were identified within 2 years, as shown by a household and institution survey. A total of 4.5% of all cases were either coprimary cases or secondary cases (*5*). Historic analysis comprising nearly 40 years based on Israeli health ministry investigations suggested that 13% of all cases were involved in outbreaks (*32*). In England and Wales, 0.5% of all cases investigated were secondary cases among close family and household contacts (*4*). Approximately 20 clusters occurred in England and Wales per year in preschool and school settings (*33*). Thus, epidemiologic surveys suggest that only a few cases are involved in clusters of IMD. This finding is supported by the results of our analysis.

The combined use of medical informatics and molecular laboratory techniques recently assisted detection of a methicillin-resistant Staphylococcus aureus outbreak in the hospital setting (*34*). The almost seamless integration of SaTScan into the database of NRZM will enable us to implement an early-warning system embedded in a geographic information system. This will support public health investigation of a serious community-acquired disease. We are currently evaluating the benefits of prospective cluster analyses and their immediate reporting to public health officials for management of IMD.
